# Are Subacute Care Patients Living Longer?

**DOI:** 10.1093/geroni/igab046.3355

**Published:** 2021-12-17

**Authors:** Nidhi Kejriwal, Samantha Tello, Brooke Davis, Mira Kubba, David Evans, Norma Gonzales, J Robert Evans

**Affiliations:** 1 University of California Los Angeles, Redlands, California, United States; 2 Western University of Health Sciences, Western University of Health Sciences, California, United States; 3 Brigham Young University, Brigham Young University, Utah, United States; 4 University of San Diego, University of San Deigo, California, United States; 5 Community Hospital of San Bernardino, Community Hospital of San Bernardino, California, United States; 6 Community Hospital of San Bernardino, evansgi, California, United States

## Abstract

In order to provide prognostic information for gerontologists who regularly counsel families, we determined to measure the longevity of subacute patients who have feeding tubes and tracheostomies. This study compares two cohorts of patients: 2002-2006 and 2015-2019. T-tests were performed to compare the total days in acute care, the total survival days, and the number of hospital admissions between the two groups. Results revealed (2002-2006, 2015-2019), some variance in the acute care days between the two groups (M= 15.4186, 21.49438) and p= .66. There is a wide difference in the total survival days between the two groups with individuals from 2015-2019 living longer than 2002-2006 (M= 229.8198, 644.0449), p< .001. However, there is no statistically significant difference in the number of hospital admissions between the two groups (M= 0.994186, 0.7752809), p= .09754. We hypothesize that advances in technology, medicine, and care over the span of 17 years contribute to increased longevity. On average, patients in the 2015-2019 group survived 414 days longer than the first group. Yet, even with such advances, more days were spent in acute care in the second group (2015-2019). Our data show subacute longevity has nearly tripled in the last decade. Although patients are living longer, they are often in a vegetative state; in most instances, there is no apparent improvement in quality of life. This study provides current data which will help gerontologists improve prognostication and allow them to form a more realistic long view of care.

